# Increased risk of chronic fatigue syndrome following psoriasis: a nationwide population-based cohort study

**DOI:** 10.1186/s12967-019-1888-1

**Published:** 2019-05-14

**Authors:** Shin-Yi Tsai, Hsuan-Ju Chen, Chi Chen, Chon-Fu Lio, Chien-Feng Kuo, Kam-Hang Leong, Yu-Ting Tina Wang, Tse-Yen Yang, Ching-Hui You, Wei-Sheng Wang

**Affiliations:** 1 Department of Laboratory Medicine, Mackay Memorial Hospital, Taipei, Taiwan; 2 Department of Medicine, Mackay Medical College, New Taipei City, Taiwan; 3 Graduate Institute of Biomedical Sciences, Graduate Institute of Long-Term Care, Mackay Medical College, New Taipei City, Taiwan; 4 Department of Health Policy and Management, Johns Hopkins University Bloomberg School of Public Health, Baltimore, MD USA; 5 College of Medicine, China Medical University, Taichung, Taiwan; 6 Management Office for Health Data, China Medical University Hospital, Taichung, Taiwan; 7 Department of Psychiatry, University of Oxford, Oxford, UK; 8 Institute of Infectious Disease, Mackay Memorial Hospital, Taipei, Taiwan; 9 Molecular and Genomic Epidemiology Center, China Medical University Hospital, Taichung, Taiwan; 10 Division of Nephrology, Department of Internal Medicine, Changhua Christian Hospital, Changhua, Taiwan; 11 National Taiwan University, College of Medicine, Taipei, Taiwan; 12 Department of Epidemiology, Harvard TH Chan School of Public Health, Boston, USA

**Keywords:** Chronic fatigue syndrome, Psoriasis, National health programs, Immune system diseases

## Abstract

**Background:**

The onset of chronic fatigue syndrome (CFS) has been shown to be associated with several immunological conditions such as infections or atopy. The aim of this study was to clarify the risk of chronic fatigue syndrome following the diagnosis of psoriasis, an immune-related dermatological disease, by analyzing the National Health Insurance Research Database of Taiwan.

**Method:**

2616 patients aged 20 years or older with newly diagnosed psoriasis during 2004–2008 and 10,464 participants without psoriasis were identified. Both groups were followed up until the diagnoses of CFS were made at the end of 2011.

**Results:**

The relationship between psoriasis and the subsequent risk of CFS was estimated through Cox proportional hazards regression analysis, with the incidence density rates being 2.27 and 3.58 per 1000 person-years among the non-psoriasis and psoriasis populations, respectively (adjusted hazard ratio [HR] = 1.48, with 95% confidence interval [CI] 1.07–2.06). In the stratified analysis, the psoriasis group were consistently associated with a higher risk of CFS in male sex (HR = 2.05, 95% CI 1.31–3.20) and age group of ≥ 60 years old (HR = 2.32, 95% CI 1.33–4.06). In addition, we discovered that the significantly increased risk of CFS among psoriasis patients is attenuated after they receive phototherapy and/or immunomodulatory drugs.

**Conclusions:**

The data from this population-based retrospective cohort study revealed that psoriasis is associated with an elevated risk of subsequent CFS, which is differentiated by sex and age.

## Background

Chronic fatigue syndrome (CFS) is a condition characterized by functional impairment, fatigue and accompanying symptoms, with a prevalence of approximately 0.1% to 2.5%, a rough estimate since diagnostics and terminology are still inconsistent. Although CFS does not cause acute injury, the condition profoundly affects those who suffer from it, as both adult and adolescent patients with CFS have a substantially lower quality of life compared with the non-CFS population [1]. CFS may be preceded by infection, exposure to environmental toxins, significant physical or emotional trauma and recent vaccination [2]. Several theoretical etiologies of CFS have been proposed, such as chronic inflammation, mitochondrial dysfunction, elevated oxidative stress, hypocortisolism and hypofunctioning hypothalamic–pituitary–adrenal (HPA) axis [3]. Most patients with autoimmune disease, like systemic lupus erythematosus and multiple sclerosis, complained about the fatigue that also correlated to the disease activity. Among these factors, the relationship between CFS and immune reactions has become one of the most intensely studied aspects of this disease. We previously reported that several immunological events, such as atopy, the reactivation of varicella-zoster virus and inflammatory bowel disease, could significantly increase the risk of CFS among the general population, further strengthening the association between CFS and a disordered immune system [4–6].

Psoriasis is a systemic immune disease that presents with dermatological as well as ophthalmologic, endocrinological, cardiovascular, and rheumatologic manifestations [7]. Systemic inflammation is one of the key symptoms of psoriasis, causing elevated circulating reactive oxygen species and other symptoms [8]. Chronic dermatological conditions such as psoriasis are considered in the sense that the innate immune system can bring about fatigue in the form of autoimmune diseases, cellular stress responses, even cancer. Many of these conditions demonstrate inflammatory or autoimmune features. From this perspective, one would expect fatigue to be common in dermatological diseases, but this aspect is often overlooked. Furthermore, the severity of psoriasis is positively related to the level of inflammatory cytokines, which provide potentially powerful targets for treating psoriasis [9, 10]. Notably, previous studies have indicated that the proportion of patients with psoriasis complaining of fatigue is larger than that of the non-psoriasis cohort, and such fatigue can be relieved by administering medication that targets inflammatory cytokines [11].

In this population-based retrospective cohort study, the increased subsequent CFS risk in patients with psoriasis was identified and analyzed by using data from the Taiwan National Health Insurance Research Database (NHIRD). Other related factors, including sex, age, comorbidity, and the severity of psoriasis were also analyzed.

## Methods

### Data sources

The NHIRD was established in 1996 and holds reimbursement claims data from the single-payer National Health Insurance (NHI) program, which was launched in 1995 and which covered approximately 99% of Taiwan’s population by the end of 2014. The NHI medical reimbursement claims database is managed by the National Health Research Institutes in Taiwan. The dataset used for this study was the Longitudinal Health Insurance Database 2000 (LHID 2000), a cohort of 1 million randomly sampled participants in the NHI system from 1996 to 2000, which includes their reimbursement information until the end of 2011. The LHID 2000 contains comprehensive information, including demographics, clinical visits, prescription details, and diagnostic codes, which is based on the International Classification of Diseases, Ninth revision, Clinical Modification (ICD-9-CM).

The reimbursement data on patient identities and institutions were scrambled cryptographically by the NHIRD to protect the privacy of the beneficiaries. The research ethics committee of the institutional review board of China Medical University, Taichung, Taiwan exempted this study from full review (CMUH104-REC2-115).

### Study population

**Fig. 1:**
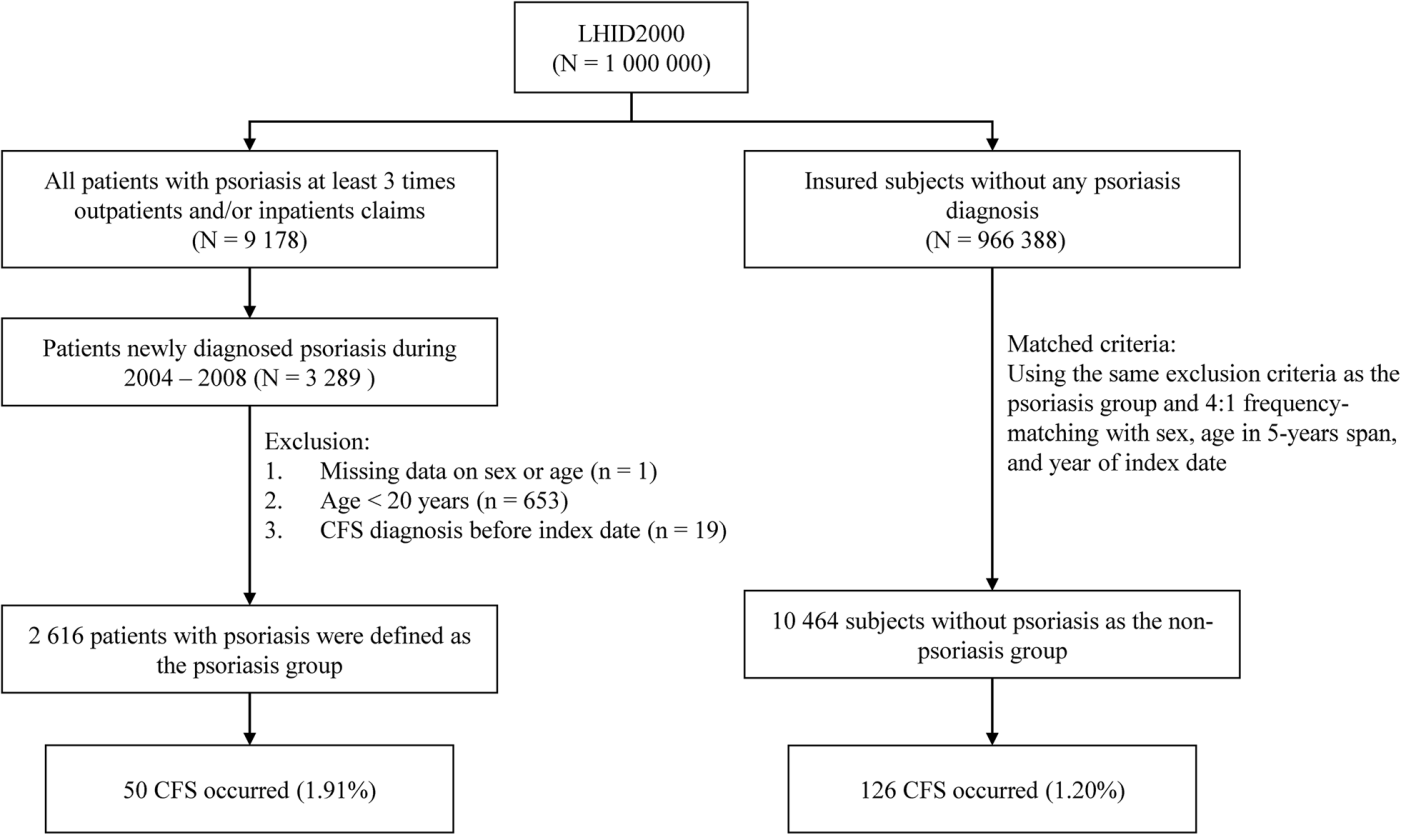
The selection process of the participants in the two study group

This population-based cohort study investigated the association of psoriasis with the risk of CFS between two groups: a psoriasis group and a non-psoriasis group. Figure 1 is a flowchart indicating how the study population was selected. We identified patients who were newly diagnosed with psoriasis (ICD-9-CM 696) during 2004–2008 as the psoriasis group; the date of psoriasis diagnosis was considered the index date. To increase the validity of psoriasis diagnoses, we selected patients who received outpatient service and/or inpatient hospitalization at least 3 times. We further excluded patients with missing information on sex or age (n = 1), aged less than 20 years of age (n = 653), and/or with a prior diagnosis of CFS (ICD-9-CM 780.71) (n = 19) from the analysis. For each patient with psoriasis, four insured participants were randomly selected from among those without psoriasis and frequency-matched by sex, age (5-year span), and index year as the non-psoriasis group, using the same inclusion criteria as that of the psoriasis group. The patient and the public were not involved in our cohort study.

#### Differentiating severity of psoriasis

Furthermore, patients with psoriasis were separated into mild psoriasis and severe psoriasis according to what treatments they received. Severe psoriasis was defined as patients receiving phototherapy (e.g., ultraviolet B (psoralen) and ultraviolet A) and/or immunomodulatory drugs (e.g., methotrexate, azathioprine, ciclosporin, oral retinoids, hydroxyurea, mycophenolate mofetil, tacrolimus, etanercept, adalimumab, and ustekinumab). Patients not receiving phototherapy and/or immunomodulatory drugs for psoriasis were classified as having mild psoriasis [12].

#### Demographic factors

Demographic factors consisted of sex and age (age groups of 20–39, 40–59, and ≥ 60 years). Medical records of comorbidities were obtained before the index date, which comprised of diabetes (ICD-9-CM 250), depression (ICD-9-CM 296.2, 296.3, 300.4, and 311), anxiety (ICD-9-CM 300.00), sleep disorder (ICD-9-CM 307.4 and 780.5), and renal disease (ICD-9-CM 580–589).

#### Follow up of participants

In our study, the primary outcome was the development of CFS (ICD-9-CM 780.71). The diagnostic criteria was based on the 1994 Fukuda definition, which requires a severe, persistent fatigue for at least 6 months with an addition of four or more symptoms, such as: unusual post-exertion fatigue, impaired memory or concentration, unrefreshing sleep, headache, muscle pain, joint pain, sore throat, and tender cervical nodes [4]. Both groups were tracked from the index date to the development of CFS, the end of 2011, or termination of the record due to either death or withdrawal from the insurance program.

### Statistical analysis

The descriptive statistics of the two groups are presented as mean and standard deviation (SD) for continuous variables and as number and percentage for categorical variables. The difference in the distribution of these statistics between the two groups was assessed using the Student’s t test for continuous variables and the Pearson’s Chi-square test for categorical variables. We calculated the incidence of CFS in the two groups by dividing the number of CFS events with the total follow-up time (per 1000 person-years). The cumulative incidence curves of CFS occurrence were generated through the Kaplan–Meier method, and the difference in the curves was assessed through a log-rank test. Univariate and multivariate Cox proportional hazards regression models were applied to assess the risk of CFS and CFS-associated risk factors. The multivariate model was adjusted for sex, age, diabetes, depression, anxiety, sleep disorder, and renal disease. Sex-, age-, and comorbidity-stratified analyses were also performed to investigate the association between psoriasis and the risk of CFS. Finally, we examined the association between different severities of psoriasis and the risk of CFS. Hazard ratios (HRs) and 95% confidence intervals (CIs) were calculated to quantify the risk of CFS.

All statistical analyses were performed using SAS 9.4 (SAS System for Windows, SAS Institute, Cary, NC, USA). The results of comparisons with a two-sided P value of < 0.05 were considered to represent statistically significant differences.

## Results

### Demographics and comorbidities according to psoriasis status

Our study included 2616 patients with psoriasis and 10,464 participants without psoriasis. The comparison between demographics and comorbidities of the psoriasis and non-psoriasis groups are presented in Table 1. The mean age of patients with psoriasis was 45.2 (SD = 17.5) years, with a mild predominance in men (approximately 54.05%). In addition, patients with psoriasis suffered from more comorbidities such as diabetes, depression, anxiety, sleep disorder, and renal disease than did the participants without psoriasis.

**Table 1:** Demographic factors and comorbidities of study participants according to psoriasis status

Variable	Non-psoriasis group (N = 10,464)		Psoriasis group (N = 2616)		P-value
	n	%	n	%	
Sex					0.99
Women	4808	46.0	1202	46.0	
Men	5656	54.0	1414	54.0	
Age, years					0.99
20–39	4620	44.2	1155	44.2	
40–59	3612	34.5	903	34.5	
≥ 60	2232	21.3	558	21.3	
Means (SD)	45.1	(17.6)	45.2	(17.5)	0.73
Comorbidity					
Diabetes	858	8.20	309	11.8	< 0.001
Depression	394	3.77	153	5.85	< 0.001
Anxiety	588	5.62	205	7.84	< 0.001
Sleep disorder	1649	15.8	515	19.7	< 0.001
Renal disease	487	4.65	177	6.77	< 0.001

*SD* standard deviation

### Cumulative incidence of CFS

**Fig. 2:**
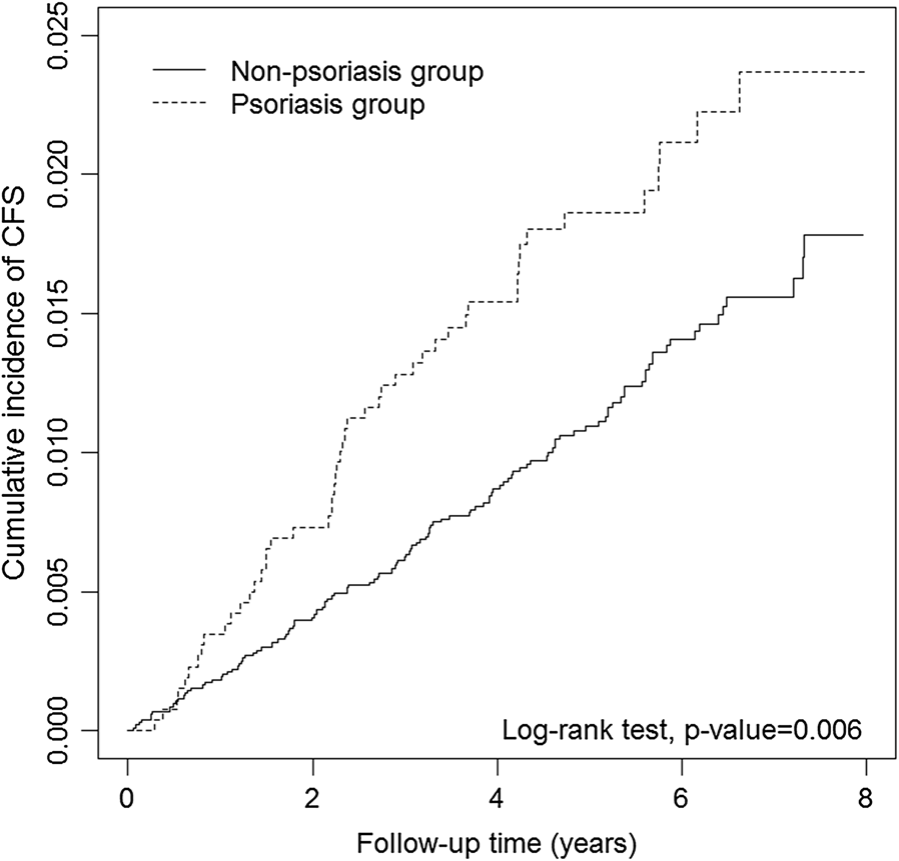
Cumulative incidence curves of chronic fatigue syndrome for groups with and without psoriasis

During the follow-up period, the number of patients diagnosed with CFS were: 50 patients with psoriasis (1.91%) and 126 participants without psoriasis (1.20%) as seen in Fig. 1. The cumulative incidence curves of CFS according to psoriasis status are illustrated in Fig. 2. A log-rank test was used to determine the cumulative incidence of CFS between the groups, showing that the cumulative incidence of CFS was significantly higher in the psoriasis group than in the non-psoriasis group (*P* = 0.006).

### Incidence density rate of CFS among psoriasis status, demographic factors, and comorbidities

The incidence density rate of CFS was higher, 3.58 per 1000 person-years among the patients with psoriasis compared with the 2.27 per 1000 person-years among participants without psoriasis. After adjustment for sex, age, and comorbidity, the adjusted HR of developing CFS was 1.48 times (95% CI 1.07–2.06) greater for patients with psoriasis than for the participants without psoriasis (Table 2). The incidence density rate of CFS is also increased with age. Compared with the younger patients (aged 20–39 years), the risk of developing CFS was 1.64-fold (95% CI 1.10–2.45) higher in those ≥ 60 years old. In the multivariate model, the risk of developing CFS was higher for the patients with a sleep disorder (adjusted HR = 2.17, 95% CI 1.53–3.08).

**Table 2:** Cox-model-measured hazard ratios and 95% confidence intervals of chronic fatigue syndrome associated with psoriasis and covariates

Variable	Event no.	Person-years	IR	HR (95% CI)	
				Univariate	Multivariate^a^
Psoriasis					
No	126	55,446	2.27	1.00	1.00
Yes	50	13,958	3.58	1.58 (1.14–2.19)**	1.48 (1.07–2.06)*
Sex					
Women	91	32,509	2.80	1.00	1.00
Men	85	36,895	2.30	0.82 (0.61–1.11)	0.86 (0.64–1.17)
Age, years					
20–39	62	31,599	1.96	1.00	1.00
40–59	60	23,974	2.50	1.28 (0.90–1.82)	1.17 (0.81–1.68)
≥ 60	54	13,832	3.90	1.99 (1.38–2.87)***	1.64 (1.10–2.45)*
Comorbidity					
Diabetes					
No	158	63,598	2.48	1.00	1.00
Yes	18	5806	3.10	1.25 (0.77–2.03)	0.83 (0.49–1.38)
Depression					
No	158	66,645	2.37	1.00	1.00
Yes	18	2760	6.52	2.75 (1.69–4.48)***	1.51 (0.89–2.57)
Anxiety					
No	153	65,385	2.34	1.00	1.00
Yes	23	4020	5.72	2.44 (1.58–3.79)***	1.30 (0.80–2.11)
Sleep disorder					
No	115	58,403	1.97	1.00	1.00
Yes	61	11,002	5.54	2.81 (2.06–3.84)***	2.17 (1.53–3.08)***
Renal disease					
No	161	66,170	2.43	1.00	1.00
Yes	15	3234	4.64	1.91 (1.13–3.24)*	1.26 (0.73–2.20)

*IR* incidence density rate per 1000 person-years, *HR* hazard ratio, *CI* confidence interval

* *P* < 0.05, ** *P *< 0.01, *** *P* < 0.001

^a^Adjusted for psoriasis, sex, age (categorical), diabetes, depression, anxiety, sleep disorder, and renal disease

When stratified by sex, the results indicated that men with psoriasis had a higher risk of developing CFS than did those without psoriasis (adjusted HR = 2.05, 95% CI 1.31–3.20; Table 3). When stratified by age group, the results indicated that the patients with psoriasis had a higher risk of CFS compared with the participants without psoriasis aged ≥ 60 years (adjusted HR = 2.32, 95% CI 1.33–4.06). When stratified by comorbidity status, the patients with psoriasis had a higher risk of CFS compared to the non- psoriasis group without comorbidity (adjusted HR = 1.66, 95% CI 1.03–2.67).

**Table 3:** Incidence density rates and hazard ratios of chronic fatigue syndrome according to psoriasis status stratified by sex, age, and comorbidity

Variables	Non-psoriasis group			Psoriasis group			Compared to non-psoriasis group	
							HR (95% CI)	
	Event no.	Person-year	IR	Event no.	Person-year	IR	Crude	Adjusted^a^
Sex								
Women	71	25,959	2.74	20	6551	3.05	1.11 (0.68–1.83)	1.07 (0.65–1.77)
Men	55	29,488	1.87	30	7407	4.05	2.17 (1.39–3.39)***	2.05 (1.31–3.20)**
Age, years								
20–39	48	25,231	1.90	14	6368	2.20	1.16 (0.64–2.10)	1.16 (0.64–2.11)
40–59	44	19,182	2.29	16	4792	3.34	1.46 (0.82–2.58)	1.26 (0.71–2.45)
≥ 60	34	11,034	3.08	20	2797	7.15	2.32 (1.34–4.04)**	2.32 (1.33–4.06)**
Comorbidity status^b^								
No	63	41,393	1.52	23	9278	2.48	1.63 (1.01–2.63)*	1.66 (1.03–2.67)*
Yes	63	14,054	4.48	27	4680	5.77	1.29 (0.82–2.02)	1.29 (0.82–2.03)

*IR* incidence density rate per 1000 person-years, *HR* hazard ratio, *CI* confidence interval

* *P *< 0.05, ** *P* < 0.01, *** *P* < 0.001

^a^Model mutually adjusted for sex, age, diabetes, depression, anxiety, sleep disorder, and renal disease

^b^Patients exhibiting diabetes, depression, anxiety, sleep disorder, or renal disease were classified as the comorbidity group

### Incidence density rate of CFS based on severity of psoriasis

Moreover, associations between different severities of psoriasis and the risk of CFS were examined, as shown in Table 4. Patients with mild psoriasis had a higher risk of CFS than did the participants without psoriasis (adjusted HR = 1.46, 95% CI 1.02–2.09). However, patients with severe psoriasis were found to have insignificant risk of developing CFS compared with the participants without psoriasis (adjusted HR = 1.59, 95% CI 0.83–3.03).

**Table 4:** Incidence density rates and hazard ratios of chronic fatigue syndrome for different severities of psoriasis

	N	Event no.	Person-years	IR	Crude HR (95% CI)	Adjusted HR (95% CI)^a^
Non-psoriasis group	10,464	126	55,446	2.27	1.00	1.00
Psoriasis group						
Mild psoriasis	2145	40	11,494	3.48	1.53 (1.07–2.19)*	1.46 (1.02–2.09)*
Severe psoriasis	471	10	2464	4.06	1.79 (0.94–3.40)	1.59 (0.83–3.03)

*IR* incidence density rate per 1000 person-years, *HR* hazard ratio; *CI* confidence interval

* *P* < 0.05

^a^Adjusted for sex, age (continuous), diabetes, depression, anxiety, sleep disorder, and renal disease

## Discussion

This population-based retrospective cohort study indicated that the psoriasis group significantly increases the incidence of CFS compared with the non-psoriasis group (Table 2, Fig. 2). Such findings are consistent with those of previous studies [13]. Furthermore, we also discovered that both male and older psoriasis patients have a higher HR of developing CFS according to subgroup analysis (Table 3), which has not been described in previous studies to our knowledge.

According to our study, men with psoriasis are more likely to be diagnosed with CFS (Table 3). Previous studies have shown that the severity of fatigue is worse in women with CFS, whereas other studies have concluded that men and women do not differ in this aspect [13, 14]. However, the prevalence of CFS is higher among both the adult and adolescent female populations (who exhibit additional symptoms such as a spastic colon and neck pain, with peak age of 30 to 50) than among men [15]. From these findings, it is suggested that the incidence of psoriasis raises the risk of CFS among men but with a lower prevalence and severity of CFS in comparison to women. The cause of this phenomenon may be sex-based differences in immune responses, which can be influenced by hormones, genetics, and other sex factors. For example, several previous studies have addressed the risk of psoriatic arthritis, which may be slightly higher in men than in women due to the potential role of hormonal influences in the pathogenesis of psoriatic arthritis- pregnancy and estrogen levels were suggested as protective factors of developing psoriatic arthritis [16]. However, these detailed mechanisms and immunomodulating effects of sex hormones require further investigations [17].

Among individuals ≥ 60 years old, psoriasis patients have an incidence rate of CFS that more than doubles in that of the non-psoriasis population, indicating that the effect of psoriasis on the etiology of CFS is significant in populations with advancing age (Table 3). The incidence rate of psoriasis has a bimodal distribution that illustrates two subtypes of psoriasis, with early-onset psoriasis being considered more genetically related [18]. Those with early- and late-onset psoriasis also have different clinical manifestations, comorbidities, reactions to treatment, and even psychological traits [19]. In addition, eruptive guttate psoriasis is often observed to follow an streptococcal infection by 2 to 3 weeks, and is believed to be an infection-induced disease [20]. CFS may be preceded by an acute or a chronic infection (viral, bacterial or parasitic) [2]. The decline of immune function in aging immune system may be contribute to development of CFS in patient with psoriasis by increased rate of infection. The mechanisms of late-onset psoriasis are poorly understood, and its association with CFS discovered in this study may provide insight for future studies.

Psoriasis is generally considered an autoimmune disease without clearly identified autoantigens and thus exhibits systemic manifestations of a dysfunctional innate and adaptive immune system, most biological agents used to treat severe psoriasis affect Th1 or Th17 pathways [21]. Innate immunity is also believed to be vital in the biological mechanisms of fatigue, with altered activities of B cells, regulatory T cells, and NK cells being identified among populations with CFS [22]. For example, activation of innate immunity can lead to increases in the expression of proinflammatory cytokines (PICs), which can not only cause inflammation, but also induce behavioral changes such as fatigue by affecting the cytokine receptors in the brain [23]. In a 2015 review I. Skoie et al. discussed the phenomenon of fatigue in psoriasis and summarized that previous clinical trials of several biologics, for example TNF-α inhibitors adalimumab or etanercept, that target the innate immunity pathway have shown reductions in the severity of fatigue in psoriasis patients. Only three of the mentioned studies utilized the Functional Assessment of Chronic Illness Therapy Fatigue subscale (FACIT-F), but all of the studies still revealed a clinically significant improvement of fatigue [11]. In our study, we compared the non-psoriasis population with patients who were and were not receiving phototherapy and/or immunomodulatory drugs (Table 4). The patients who did not receive phototherapy and/or immunomodulatory drugs had significantly higher HRs of CFS than did the patients who received these interventions, further confirming the effect of such treatments on fatigue and indirectly indicating the role of immunity in the etiology of CFS.

One of the most researched types of CFS pathophysiology is the dysregulation of the HPA axis. Abnormal adrenocortical activity has been reported among CFS patients since 1981 [24]. We previously reported that burn injury can disturb HPA axis and increase risk of subsequent CFS [25]. The HPA axis provides the body with the capability to respond to stress, which is a self-regulated feedback system that maintains homeostasis [26]. This feedback system includes the paraventricular nucleus of the hypothalamus, pituitary, and adrenal glands. Positive feedback from the hypothalamus to the adrenal gland provided by mediating hormones such as the corticotropin-releasing hormone, arginine vasopressin, and adrenocorticotropic hormone (ACTH) stimulates the cortisol secretion of the adrenal gland. However, circulating cortisol suppresses the secretion of upstream hormones through the binding of the mineralocorticoid receptor (MR) and glucocorticoid receptor (GR) implementation of the feedback loops of the HPA axis [27]. Several reviews have suggested that the abnormality of the HPA axis might be a common feature among the CFS population [28]. For example, hypocortisolaemia [24], loss of the diurnal peak of ACTH and cortisol levels [24, 28], and blunted responsiveness of the HPA axis during a challenge test have all been reported [29]. Some authors have hypothesized that the fundamental cause of the impaired HPA response of CFS might be over-activity of the GR and MR, which leads to increased suppression of the hypothalamus and anterior pituitary components [30]. Although typical symptoms of CFS can be presented in those with hypocortisolemia, the altered HPA-axis function potentially reduces the capacity of HPA hormones to counteract the immune system. As a result, an inflammatory response may be easily triggered by slight stressors [31]. Subsequent PIC storms, such as secretion of IL-6, have been correlated with sustained fatigue and other symptoms that are exhibited by CFS patients [32, 33]. Immune activation markers of CFS include increased levels of PICs such as tumor necrosis factors TNFα, IL-6, IL-1β [34]. Recent studies have suggested that levels of IL-1 and TNFα have a significantly positive correlation with fatigue, autonomic symptoms, and flu-like symptoms [35]. Some clinical trials of treating CFS with biological agent have been emerging, Rituximab has had the best improvement rates in chronic fatigue syndrome (CFS) in randomised placebo-controlled and open studies [36, 37]. Since the diagnostic criteria in CFS is still debatable, HPA-axis dysregulation seems to play a crucial role in the pathophysiology of CFS. Due to the varied and often debilitating manifestations displayed by these patients, the etiology of CFS is most likely multifactorial encompassing several body systems, diseases, or even genetic predisposition and thus, finding a treatment that works for every CFS patient is a challenging task. Multidisciplinary rehabilitation treatment is effective at reducing long-term fatigue severity in patients with CFS [38, 39], the intent is to build an increased awareness and consciousness of healthy bodily symptoms and their relation to physical function, psychological wellbeing, and social interaction. Yet with a renewed interest in this previously rather shunned condition, setting a clear diagnostic criterion has become a priority, with the development of what might be the first diagnostic test under way [40].

Psoriasis is a chronic inflammatory disease that increases the prevalence of a variety of psychosomatic disorders [41]. In 1985, Arnetz et al. demonstrated that after a stress test, lower cortisol levels were seen in psoriasis patients than in the control (non-psoriasis) population [42]. Another study of 102 psoriasis patients’ salivary cortisol values revealed that bedtime cortisol levels were correlated with psoriasis severity, as measured by the Psoriasis Area Severity Index (PASI) [43]. Other researchers have observed hypocortisolemia in psoriasis patients in high-stress populations [44]. All of these results indicate a correlation between psoriasis and cortisol levels, suggesting that an HPA-axis dysfunction might play a crucial role in psoriasis patients when their bodies are managing stress [42]. Subsequent release of PICs could aggravate psoriasis [45], and theoretically, typical symptoms of CFS. Although the exact mechanism of the impairment of the HPA-axis function in psoriasis and CFS patients is not yet clear, some CFS patients may benefit from therapy that is intended to restore HPA-axis function. Low-dose oral hydrocortisone supplement therapy was administered in an RCT study, which showed improvement of CFS symptoms in the experimental group. However, suppression of adrenal glucocorticoid responsiveness limited the practical use of this therapy for treating CFS [46]. In future studies, more applicable rehabilitation programs or pharmacologic agents that recover the responsiveness of the HPA-axis in CFS patients merit exploration.

Chronic fatigue syndrome patients have clinical depression and/or anxiety [47]. Psychosocial stress and mental illness are comorbidities of psoriasis, including anxiety disorder, depression, social phobia, alcoholism, sexual dysfunction and somatoform symptoms. However, the neurobiological, psychological and social interactions in patients with psoriasis and potential psychological and mental comorbidities that have not been fully understood yet [48, 49]. Several inflammatory and pathogenic pathways in depression have been proposed, such as reduced brain monoaminergic transmission (e.g., serotonin, norepinephrine), increased proinflammatory cytokines (e.g., IL-1, IL-6, IL-17, TNFα), reduced neurotrophic factors, elevated oxidative stress and dysregulation of HPA-axis, which are similar to CFS [50]. In addition, some evidence showed biologic DMARDs (e.g., methotrexate), which is used to treat severe psoriasis, had the highest rates of depression, anxiety and suicidal ideation [51]. The psychosocial and mental correlations between CFS and psoriasis are necessary to be determined in future studies.

Since 99.9% of Taiwan’s population is currently enrolled, coverage of the Taiwan NHI program is highly comprehensive. This high percentage of enrollment minimized the selection bias in our study. Furthermore, we attest to the reliability and accuracy of the diagnoses by clinical physicians due to the scrutiny of peer review and medical reimbursement specialists for insurance claim purposes.

Our study had some limitations. First, the complications of both psoriasis and CFS and their severity (based on PASI), have not been considered in this study because of the limited information gathered from the NHIRD. Whether the severity of psoriasis and the risk of CFS are positively associated has yet to be determined. Second, patients’ histories (including symptoms, occupation status, family history), serum laboratory data, and related clinical variables were unavailable because of the anonymity of the data from the NHIRD, which prevented our group from analyzing the relationships among psoriasis, CFS, serum C-reactive protein levels, and other detailed data. Third, the studied population was mainly composed of East Asians living in Taiwan. Whether ethnic or geographic discrepancies exist within this population requires further examination. In addition, the relationships between CFS and different subtypes or manifestations of psoriasis (such as psoriatic arthritis) which have not been discussed, will be the focus of our future study. Fourth, the duration of our study was between 2004 and 2008, and although multiple diagnostic criteria were developed for CFS in recent years, we still chose to include the participants based on the widely- accepted 1994 Fukuda definition. Thus, patients who were diagnosed with CFS by other criteria were not included in our study.

## Conclusion

Psoriasis significantly increases the risk of CFS, especially in men and the aging population. These increased risks can be attenuated in patients who receive phototherapy or immunomodulatory drugs.

## Data Availability

The data underlying this study is from the National Health Insurance Research database (NHIRD). Interested researchers can obtain the data through formal application to the Ministry of Health and Welfare, Taiwan.

## Abbreviations

CFS: chronic fatigue syndrome
HPA axis: hypothalamic–pituitary–adrenal axis
NHIRD: National Health Insurance Research Database
LHID: Longitudinal Health Insurance Database
SD: standard deviation
HRs: hazard ratios
CIs: confidence intervals
PICs: proinflammatory cytokines
ACTH: adrenocorticotropic hormone
MR: mineralocorticoid receptor
GR: glucocorticoid receptor
TNFα: tumor necrosis factor α
IL-1: interleukin-1
IL-6: interleukin-6
IL-1β: interleukin-1β
